# Four novel genes associated with longevity found in Cane corso purebred dogs

**DOI:** 10.1186/s12917-022-03290-9

**Published:** 2022-05-19

**Authors:** Evžen Korec, Lenka Ungrová, Jiří Hejnar, Adéla Grieblová

**Affiliations:** 1 Zoologická zahrada Tábor a.s., Dukelských Hrdinů 19, 170 00 Prague 7, Czech Republic; 2 Institute of Molecular Genetics of the Czech Academy of Sciences, Vídeňská 1083, 142 20 Prague 4, Czech Republic

**Keywords:** Longevity-associated genes, GWAS, Cane corso Italiano dog, Extending lifespan, Longevity testing

## Abstract

**Background:**

Longevity-related genes have been found in several animal species as well as in humans. The goal of this study was to perform genetic analysis of long-lived Cane corso dogs with the aim to find genes that are associated with longevity.

**Results:**

SNPs with particular nucleotides were significantly overrepresented in long-lived dogs in four genes, *TDRP*, *MC2R*, *FBXO25* and *FBXL21*. In *FBXL21*, the longevity-associated SNP localises to the exon. In the FBXL21 protein, tryptophan in long-lived dogs replaced arginine present in reference dogs.

**Conclusions:**

Four SNPs associated with longevity in dogs were identified using GWAS and validated by DNA sequencing. We conclude that genes *TDRP*, *MC2R*, *FBXO25* and *FBXL21* are associated with longevity in Cane corso dogs.

**Supplementary Information:**

The online version contains supplementary material available at 10.1186/s12917-022-03290-9.

## Background

Longevity and lifespan extension represent a subject of interest and research in various organisms from yeasts to vertebrates including humans [1–3]. According to recent studies, extended lifespan is a polygenic trait influenced not only genetically, but also by epigenetic, environmental, and behavioural factors [4–6]. Heritability of extended lifespan varies from 20 to 35% in humans [5]. So far, 33 genes have been associated with longevity in mice and thanks to genetic mapping, next generation sequencing and genome-wide association studies (GWAS), dozens of candidate genes and hundreds of associated single nucleotide polymorphisms (SNPs) were described in humans [2, 5–7]. Nevertheless, the recent knowledge does not allow any significant extension of the lifespan in humans and animals.

In the last years, GWAS have been done in dogs with the aim to map genetic loci associated with general or breed-specific diseases [8–10] and eliminate the risk alleles. In addition to that, understanding the genetic component of longevity in dogs is also important for selective breeding. Extended lifespan, especially in large dog breeds with a generally short lifespan, is a desirable goal. Interestingly, long co-evolution of dogs and humans led to sharing not just their lifestyle, but also common diseases such as diabetes [11, 12]. In combination with artificial selection, genetic diversity among breeds and health care comparable to humans, dogs represent an interesting model organism for longevity research.

So far, only a few studies have investigated the genetic background of the dog longevity. A whole-genome sequencing study of two extremely old dogs showed multiple potential loci that could be closely associated with longevity [13]. Another study focusing on genes associated with cancer mortality and longevity also suggests the need for further research in this field [14]. Such results are challenging for further identification of genes and loci associated with longevity in dogs.

This study focuses on finding genes associated with longevity in purebred Cane corso dogs. Cane corso is a large Molossian breed with a median lifespan of merely 9.29 years [15], which exemplifies the fact that the lifespan of large dog breeds is significantly shorter compared to small breeds [16]. Shorter lifespan in larger breeds is often caused by gastrointestinal and musculoskeletal diseases, such as canine hip dysplasia (CHD) [16–19]. The most frequent causes of death, such as cancer, cardiac and urological diseases, have been described using statistical methods. Young dogs commonly died from gastrointestinal disorders or as a result of an infection, whereas older dogs suffered from neurological and neoplastic diseases [17]. Balanced nutrition and generally good body condition, particularly appropriate physical activity and preventing obesity is important for extending the lifespan in large dog breeds. Lifelong maintenance of lean body mass and attenuated accumulation of body fat were key factors in achieving an exceptional lifespan in Labrador retrievers [20].

The inheritance of CHD [21–23] and coat colour [24] have been described in the breed Cane corso. This study identifies four candidate genes associated with longevity in the Cane corso dogs using the GWAS method and subsequent confirmative sequencing.

## Methods

Buccal swab samples from purebred Cane corso dogs were collected during the years 2016–2020. The health status of the dogs was not evaluated. For the purpose of this research, samples were divided into two groups defined by the age of the dogs examined. For the reference group, we sampled dogs at the age between 2 and 9 years. The group of long-lived dogs contained samples from individuals older than 12 years, which were considered as long-lived dogs according to a previous study of the Cane corso breed longevity [15]. 12 years as a limit for the category of long-lived dogs was chosen because 12 years is in the fourth quartile of the distribution pattern for age at death of Cane corso dogs and the probability that the dogs from the reference group would reach this age is less than 7% [15]. Overall, 20 samples of long-lived dogs and 20 samples of reference dogs were used for this study. Since dogs from the reference group could possibly be long-living, monitoring of these dogs will continue to confirm our results.

DNA was isolated from buccal swab samples using a Qiagen DNeasy Blood & Tissue Kit and standard phenol-chloroform DNA isolation protocol. DNA was eluted in 15 to 50 μl elution solution. Concentration and purity of isolated DNA was checked using a spectrophotometer. The required length of 5000 base pairs (bp) for the SNP genotyping was checked in 2% agarose gel. Suitable samples were diluted or concentrated to the required concentration of DNA for 20–30 ng/μl. Out of all the isolated samples, 9 samples from the long-lived group and 15 reference samples had sufficient concentration and bp length and were suitable for SNP genotyping. Samples were genotyped using Illumina CanineHD Beadchip at Neogen laboratory, 4131 N. 48th St. Lincoln, NE 68504, USA. This chip allows analysis of 172,115 SNPs.

Genome wide association study (GWAS) was performed on 24 samples (9 long-lived and 15 reference) suitable for SNP genotyping to identify candidate longevity-associated SNPs. Statistical significance and overrepresentation of candidate SNPs in long-lived dogs were then validated by sequencing whole sample panel of 40 dogs (20 samples of long-lived dogs and 20 samples of reference dogs).

Statistical analysis and necessary steps that preceded the association analysis were performed using PLINK v1.90b6.16 [25]. Received data were checked according to commonly used quality parameters. Firstly, SNPs that were missing in more than 10% (geno 0.1) of the samples were excluded from further analysis. Of the rest of the markers, those that were missing in more than 1% (geno 0.01) of all samples were also excluded. All samples kept for further analysis have more than 95% of SNP markers genotyped (mind 0.05). After this first step of data cleaning, 71,660 variants and 24 dogs (9 long-lived and 15 reference) passed the data clean up. SNPs with minor allele frequencies lower than 5% (maf 0.05) were also excluded from the association analysis. Although the number of samples was low and the model organism was a highly inbred domestic animal, markers that did not fit in Hardy Weinberger Equilibrium in threshold 0.0001 were excluded. 47,915 SNP variants and 24 (9 long-lived and 15 reference) dogs passed for further genome-wide association analysis. Association analysis was performed using 1df chi-square allelic test. *P*-values were adjusted by Benjamini-Hochberg correction [26]. Principal component analysis (PCA) vectors and values were also exported using PLINK.

PCA plot, Manhattan plot for visualization of the association analysis and qqplot of the association analysis were constructed in R Studio [27] using packages ggplot2 [28], qqman [29] and lattice [30] that are available on the CRAN repository. All analyses and plotting were done using R programming language version 4.1.3 [31].

According to the result of GWAS, genomic position of all candidate SNPs was checked in the CanFam 3.1 reference genome. Seven candidate SNPs and their close surroundings, located in seven genes, were PCR amplified and sequenced in 40 samples including those used for GWAS. Samples were sequenced in SEQme s.r.o., 26,301 Dobris, Czech Republic.

Statistical significance of the distribution of genotypes from all sequenced samples within the long-lived and reference groups was tested in R Studio [27] using Fisher’s exact test [32].

Genomic region figures were created using NCBI Genome Data Viewer [33] and were simplified for easier understanding.

## Results

### SNP genotyping and GWAS

**Fig. 1:**
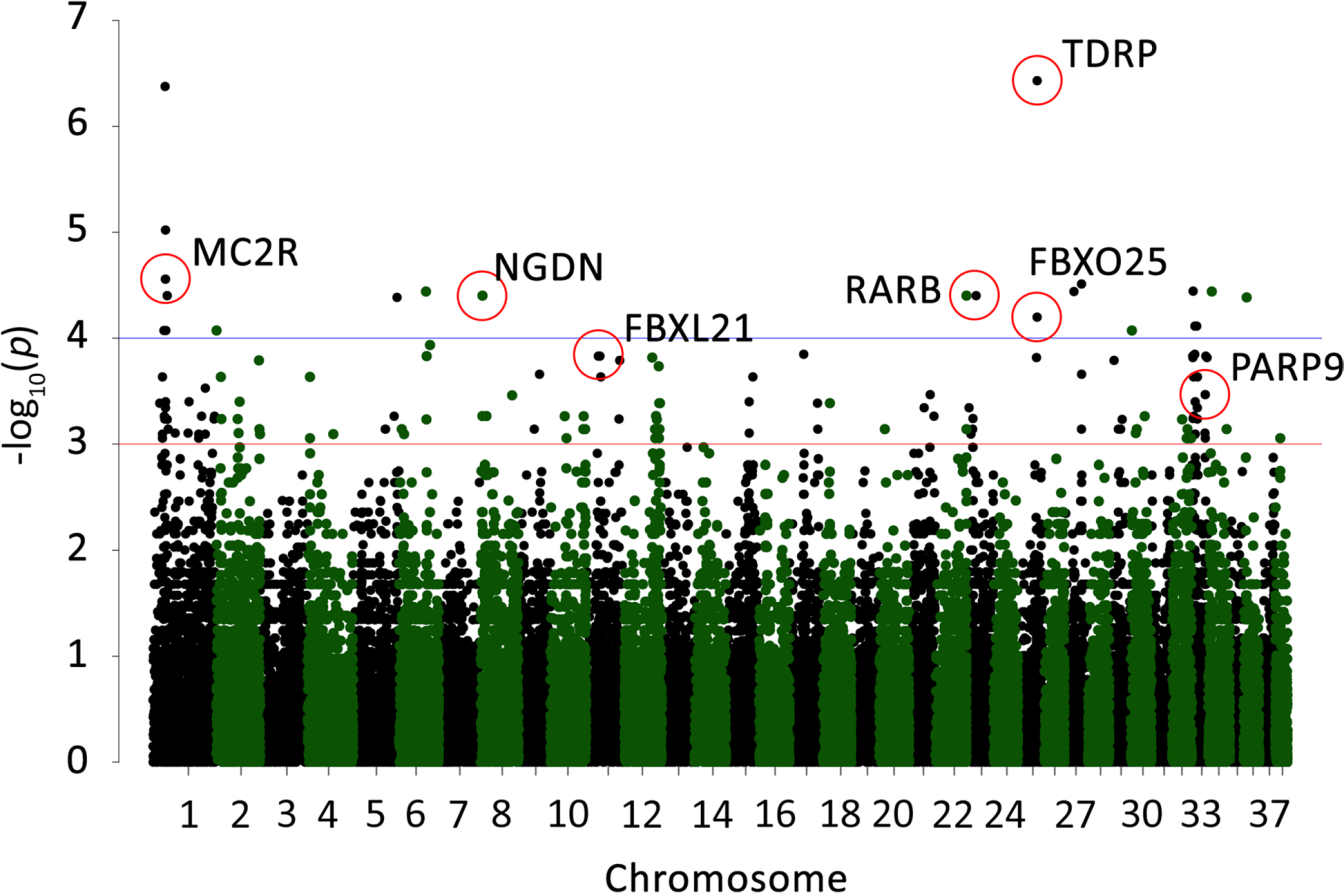
Manhattan plot of the GWAS results. Blue line represents a significance threshold (1.0e-04). Red line represents a significance threshold (1.0e-03). SNPs chosen for further analyses are circled

Overall, 24 SNPs passed through the set significance threshold (1.0e-04) (Fig. 1). The lower significance threshold was chosen because GWAS served only as a search tool to find candidate SNPs that were subsequently sequenced using a larger sample set. Eleven SNPs that passed the significance threshold were localized within the described genes in the CanFam 3.1 reference genome. Four SNPs located in the intron region of the genes and one SNP located in the 3’UTR part of one gene were selected for sequencing in the entire sample panel. Next, two SNPs located in the exon region with the highest *P*-value were also sequenced in the entire sample panel. Results from the association analysis for the seven selected SNPs and their position in particular genes are shown in Table 1.

**Table 1:** Table of GWAS results for 7 candidate SNPs sorted by the highest *P*-value. *P*-value, unadjusted *P*-value; *P*-value_BH, *P*-value adjusted according to Benjamini-Hochberg correction; CHR, chromosome; SNP, single nucleotide polymorphism; BP, genomic position (base-pair); A1, minor allele; F_A, frequency of minor allele in long-lived group; F_U, frequency of minor allele in reference group; A2, major allele; CHISQ, basic allelic chi-square test; OR, estimated odds ratio

Gene	***P***-value	***P***-value_BH	Position	CHR	SNP	BP	A1	F_A	F_U	A2	CHISQ	OR
*TDRP*	3.705e-07	0.01006	3’UTR	25	BICF2P365740	37,486,799	T	0.7222	0.03333	C	25.84	75.4
*MC2R*	2.772e-05	0.09404	intron	1	BICF2P1140956	24,364,734	G	0.7778	0.1667	A	17.57	17.5
*NGDN*	3.969e-05	0.09404	intron	8	BICF2P384545	3,705,090	C	0.6111	0.06667	T	16.89	22
*RARB*	3.969e-05	0.09404	intron	23	BICF2G630382633	18,647,529	C	0.6111	0.06667	T	16.89	22
*FBXO25*	6.334e-05	0.138	intron	25	BICF2P999174	37,425,075	A	0.4444	0	G	16	NA
*FBXL21*	1.478e-04	0.1766	exon	11	chr11_23,852,542	23,852,542	T	0.6667	0.1333	C	14.4	13
*PARP9*	3.413e-04	0.2636	exon	33	chr33_25,671,811	25,671,811	A	0.7222	0.2	C	12.83	10.4

### Sequencing

**Fig. 2:**
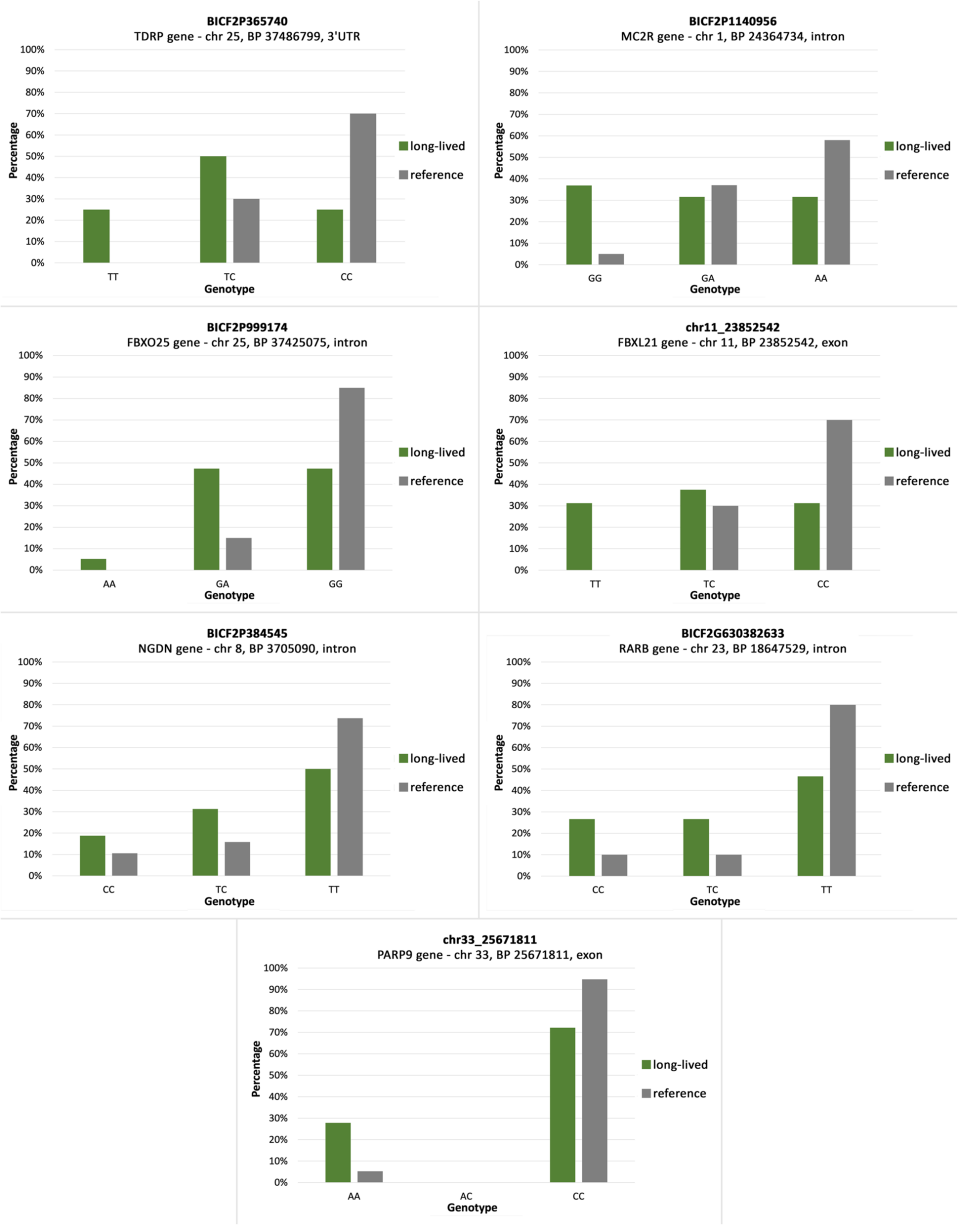
Distribution of genotypes in the SNPs of the investigated genes after sequencing of the whole sample panel. Green, long-lived group; grey, reference group

After sequencing of the whole sample panel of 40 dogs (20 long-lived, 20 reference), differences were found between long-lived and reference samples (Fig. 2).

**Fig. 3:**
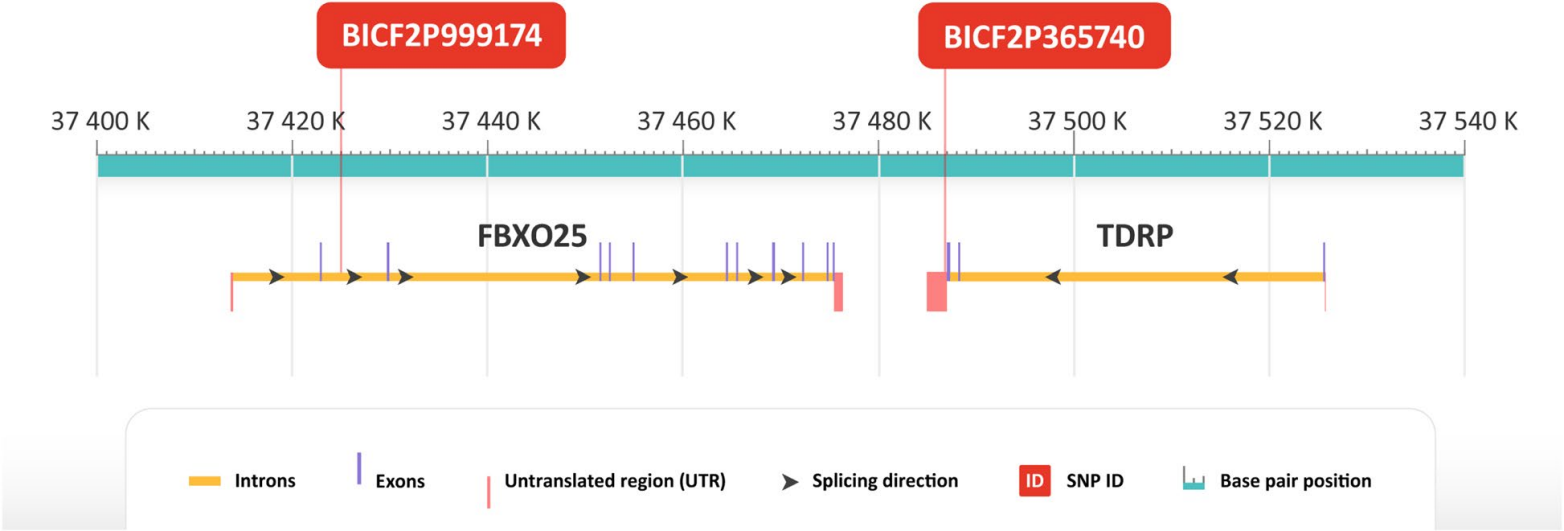
Genomic region of chromosome 25 showing longevity-associated SNPs in closely located genes FBXO25 and TDRP

In the most significant SNP BICF2P365740 (CHR 25, BP position 37,486,799) according to GWAS, located in the 3’UTR part of the *TDRP* gene (Fig. 3), nucleotide T was considered as an allele associated with longevity. After sequencing of the whole sample panel, 25% of the long-lived group were homozygous with genotype TT compared to 0% in the reference group. 50% of the long-lived group were heterozygous with genotype TC compared to 30% of the reference group. Only 25% of the long-lived group were homozygous with genotype CC compared to 70% of the reference group (Fig. 2). Association of allele T with longevity in homozygous and heterozygous form was significant in Fisher’s exact test (*p* = 0.04712). Genotype TT was proved to be associated with longevity, as it was found exclusively in long-lived dogs.

Next to the *TDRP* gene on chromosome 25, *FBXO25* gene is located. In the second intron of the *FBXO25*, SNP BICF2P999174 (CHR 25, BP 37425075) is located (Fig. 3). According to GWAS, allele A was considered as an allele associated with longevity (Fig. 1). After sequencing of the whole sample panel, 5% of the long-lived group were homozygous with genotype AA compared to 0% in the reference group. 47% of the long-lived group were heterozygous with genotype GA compared to 15% of the reference group. 47% of the long-lived group were homozygous with genotype GG compared to 85% of the reference group (Fig. 2). Association of allele A with longevity in either homozygous or heterozygous form was significant in Fisher’s exact test (*p* = 0.04074). Genotype AA was proved to be associated with longevity, as we have found it exclusively in long-lived dogs.

**Fig. 4:**
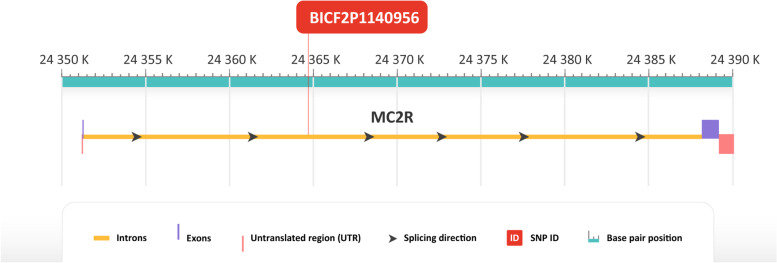
Genomic region of chromosome 1 including MC2R gene with SNP associated with longevity

The second most significant SNP BICF2P1140956 (CHR 1, BP 24364734) according to GWAS is located in the first intron of the *MC2R* gene (Fig. 4). For this position, nucleotide G was considered as an allele associated with longevity (Fig. 1). After sequencing of the whole sample panel, 37% of the long-lived group were homozygous with genotype GG compared to 5% in the reference group. 32% of the long-lived group were heterozygous with genotype GA compared to 37% of the reference group. 32% of the long-lived group were homozygous with genotype AA compared to 58% of the reference group (Fig. 2). Association of allele G with longevity in homozygous and heterozygous form was significant in Fisher’s exact test (*p* = 0.01966).

**Fig. 5:**
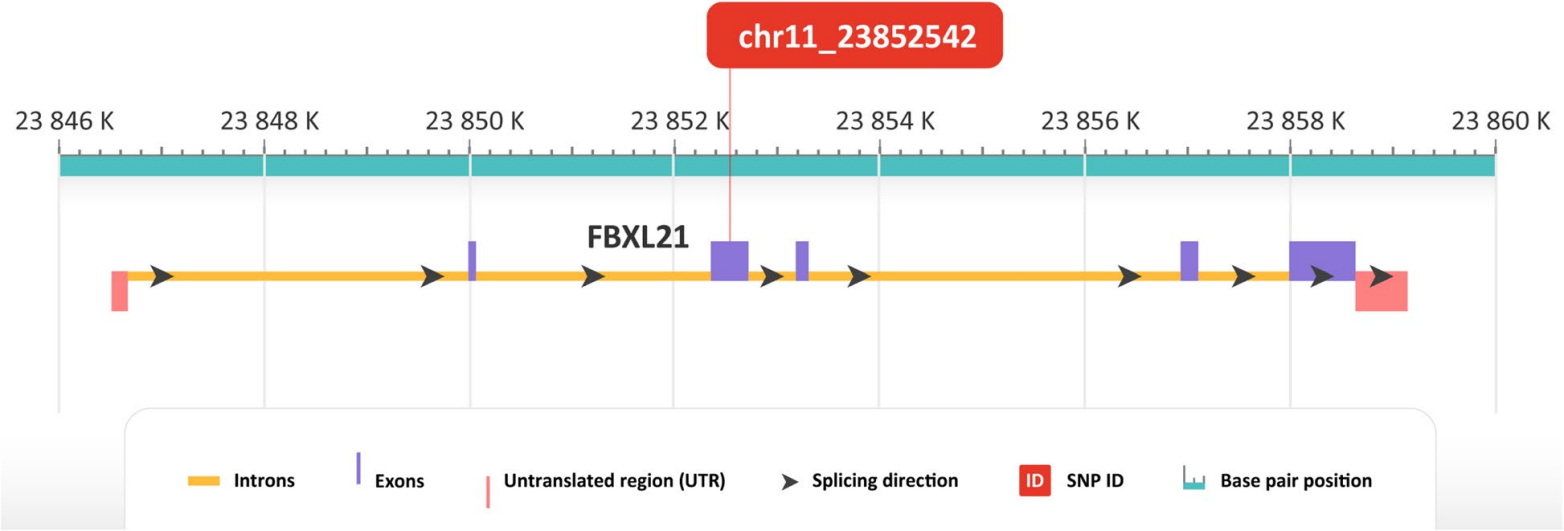
Genomic region of chromosome 11 including FBXL21 gene with SNP associated with longevity

For SNP chr11_23,852,542 (CHR 11, BP 23852542), located in the third exon of the *FBXL21* gene (Fig. 5), nucleotide T was considered as an allele associated with longevity (Fig. 1). After sequencing of the whole sample panel, 31% of the long-lived group were homozygous with genotype TT compared to 0% in the reference group. 38% of the long-lived group were heterozygous with genotype TC compared to 30% of the reference group. 31% of the long-lived group were homozygous with genotype CC compared to 70% of the reference group (Fig. 2). Association of allele T with longevity in either homozygous or heterozygous form was significant in Fisher’s exact test (*p* = 0.0111). With this nucleotide substitution from C to T in the SNP position, amino acid arginine is replaced by tryptophan. Genotype TT was proved to be associated with longevity and we observed it exclusively in long-lived dogs.

*P*-value adjusted according to Benjamini-Hochberg correction in GWAS was statistically significant only for the TDRP gene. For MC2R, FBXO25 and FBXL21, the adjusted *P*-value was not statistically significant.

Statistical evaluation using the Fisher’s exact test performed on a larger set of sequenced samples showed statistically significant differences. Therefore, we believe that the MC2R, FBXO25 and FBXL21 genes are associated with longevity.

For SNP chr33_25,671,811 (CHR 33, BP 25671811), located in the second exon of the *PARP9* gene, nucleotide A was considered as an allele associated with longevity (Fig. 1). Association of allele A with longevity was not significant in Fisher’s exact test (*p* = 0.08968). For SNP BICF2P384545 (CHR 8, BP 3705090), located in the first intron of the *NGDN* gene, nucleotide C was considered as an allele associated with longevity (Fig. 1). Association of allele C with longevity was not significant in Fisher’s exact test (*p* = 0.3044). Results for SNP BICF2G630382633 (CHR 23, BP 18647529), located in the third intron of the *RARB* gene, suggested that nucleotide C was considered as an allele associated with longevity (Fig. 1). Association of allele C with longevity was not significant in Fisher’s exact test (*p* = 0.1523). No statistically significant association with longevity was demonstrated for this gene. We did not find enough evidence to suggest that these three genes are associated with longevity in Cane corso dogs.

## Discussion

Longevity-associated genes have been found in several animal species as well as in humans [1–3]. Such genes have not yet been described in dogs. In this study, we describe four SNPs that are associated with longevity in the Cane corso breed. We used GWAS as a prediction tool for selecting candidate SNPs for further sequencing in larger sample set. Even though the size of the GWAS dataset was quite small and none of the SNPs reached the genome-wide significance threshold, we sequenced selected candidate SNPs in 20 samples of long-lived dogs and 20 samples of reference dogs including those used for GWAS to validate our findings.

Thanks to the sequencing of larger sample size, we were able to find 4 genes in which particular genotypes were significantly overrepresented in long-lived dogs.

According to GWAS and DNA sequencing the most significant SNP associated with longevity was located in the 3’UTR of the *TDRP* gene. *TDRP* was not previously described as associated with longevity, but it is associated with spermatogenesis and sperm motility [34]. Untranslated regions at the 3′ end can play an important role in the regulation of translation and mRNA stability [35]. The role of 3’UTR SNPs in longevity was also previously described in humans [36]. Regulation of gene expression could play an important role in longevity and thus it should be further investigated. On the chromosome 25 close to the *TDRP* gene, we found another SNP significantly associated with longevity in an intron of the gene *FBXO25*. *FBXO25* plays a role in promoting tumour growth [37, 38]. Finding two significant SNPs in closely located genes suggests that this region on chromosome 25 can play an important role in longevity of Cane corso dogs.

Another significantly associated SNP is located in intron of *MC2R* (adrenocorticotropic hormone receptor) gene. Change in expression of the receptor could possibly cause a change in regulation of the secretion of adrenocorticotropic hormone which often leads to hyperadrenocorticism and adrenocortical tumor. Both of those conditions lead to earlier death in affected dogs [39]. *MC2R* was also previously described as associated with longevity in humans [6]. These results suggest that the association of this gene with longevity could be species independent.

Determining the causal relationship between a particular nucleotide substitution and longevity can be crucial in identifying the predisposition to longevity at the genetic level. From this point of view, SNPs located in gene exons could be of greatest importance. We detected one gene where the SNP associated with longevity is located in an exon. In the *FBXL21* gene, a nucleotide substitution leads to an amino acid change resulting in tryptophan presence in long-lived dogs, while arginine is produced in reference dogs. *FBXL21* has an important role in the oscillation of the circadian clock [40]. Optimal circadian rhythms seem to have an influence on aging [41]. Circadian rhythms have most often been described in terms of their phases and amplitudes, and how these respond, in both health and disease, to a single exposure to synchronizers. Daily fluctuation of several physiological functions, which has been studied in dogs, demonstrates a daily rhythmicity [42, 43]. Substitution of one amino acid can lead to a change in the structure of the protein produced, which can then cause a change in its function. Analysis of the structure of such proteins will be the subject of our further research.

All the described genes could have a direct influence on extending lifespan. Three of the investigated genes, *PARP9*, *NGDN* and *RARB*, were not significantly associated with longevity in the Cane corso breed.

Testing the SNPs in genes that are associated with longevity determined in this study could allow prediction of the possible longevity of the tested dog. Crossing dogs with longevity potential could allow breeding of long-lived dog lineages which is very important for every breeder and dog owner. The development of such test will be the subject of our further study. We however need to confirm our results in a longitudinal study of additional dogs before the longevity-associated SNPs could be used as breeding markers.

## Conclusions

Four SNPs shown to be associated with longevity in Cane Corso dogs were identified using GWAS and DNA sequencing. Genes *TDRP*, *MC2R*, *FBXO25* and *FBXL21* are associated with longevity in Cane corso dogs.

## Supplementary Information

**Additional file 1.**

## Data Availability

The datasets used in this study are available from https://www.ebi.ac.uk/eva/?Study-Browser&browserType=sgv, project accession: PRJEB51024.

## Abbreviations

CHD: Canine hip dysplasia
GWAS: Genome-wide association study
SNP: single nucleotide polymorphism
