# Supplementary material for: Four novel genes associated with longevity found in Cane corso purebred dogs
Source: BMC Vet Res. 2022 May 19;18:188. doi: 10.1186/s12917-022-03290-9 (PMC9118790; doi:10.1186/s12917-022-03290-9)
Supplement: Supplementary file 1 — Additional file 1. [file 12917_2022_3290_MOESM1_ESM.docx]

Appendix

**Table A1.** Table of sampled dogs and their assignment to the groups. Y= years, m= months.

| **Name** | **Group** | **Sex** | **Date of birth** | **Age of death** |
| --- | --- | --- | --- | --- |
| Aisha Blue Princess | long-lived | F | 30.08.2008 |  |
| Appia Fosso Corno | long-lived | F | 14.08.2006 |  |
| Aragorn The Best Caco | long-lived | M | 02.02.2005 | 13y, 10m |
| Arena | long-lived | F | 10.05.2005 |  |
| Avalanche Crnobog | long-lived | M | 05.06.2006 |  |
| Azzura Giantpaw's | long-lived | F | 15.12.2007 |  |
| Cesare | long-lived | M | 05.04.2004 | 12y, 9m |
| Cora | long-lived | F | 01.07.2005 |  |
| Dominika Atison | long-lived | F | 03.10.2008 |  |
| Edgar Georg Cane Corso | long-lived | M | 30.10.2003 | 13y, 11m |
| Fee dei Fontanili | long-lived | F | 11.10.2007 |  |
| Gera | long-lived | F | 05.04.2006 |  |
| Gloria Abdanka Cember | long-lived | F | 17.09.2005 | 12y, 3m |
| Greta Corso of Bajer | long-lived | F | 12.08.2006 |  |
| Hihankara's Eleganza | long-lived | F | 08.08.2004 |  |
| Irvin Corso pod Zoborom | long-lived | M | 28.04.2007 |  |
| Nico | long-lived | M | 16.05.2008 | 12y, 7m |
| Samba | long-lived | F | 25.04.2007 |  |
| Tiana Bailando Con Lobos | long-lived | F | 05.02.2002 | 16y, 3m |
| Your Majesty del Orte | long-lived | F | 12.09.2008 |  |
| Aristocrat Korec Corso | reference | M | 13.02.2017 |  |
| Blú Petto Largo | reference | M | 19.10.2015 |  |
| Borgie Korec Corso | reference | F | 22.09.2017 |  |
| Boy Korec Corso | reference | M | 22.09.2017 |  |
| Brittany Korec Corso | reference | F | 22.09.2017 |  |
| Bucci of Michalec | reference | M | 31.08.2014 |  |
| Ceres Conny Gemma Nera | reference | F | 10.07.2017 |  |
| Chalu Gucci Angelo Custode | reference | M | 15.05.2017 |  |
| Dakar Gemma Nera | reference | M | 02.10.2017 |  |
| Elisa Berren Corso | reference | F | 08.08.2015 |  |
| Faith Petto Largo | reference | F | 08.10.2016 |  |
| Infinity Geraldino | reference | F | 08.09.2015 |  |
| Ivan II Petto Largo | reference | M | 05.09.2017 |  |
| Kate Atison | reference | F | 12.09.2012 |  |
| Koleta Atison | reference | F | 12.09.2012 |  |
| Madeira Petto Largo | reference | F | 27.08.2018 |  |
| Samm Corso z Jasnej hviezdy | reference | M | 30.11.2017 |  |
| Sofie Atison | reference | F | 19.11.2014 |  |
| Xavera Atison | reference | F | 31.10.2016 |  |
| Zeta Crazy Clan | reference | F | 14.07.2016 |  |

**Table A2.** Table of genotypes for each sample and each of the sequenced SNP in selected genes.

| **Name** | **Group** | **Genotype *TDRP*** | **Genotype *MC2R*** | **Genotype *FBXO25*** | **Genotype *FBXL21*** | **Genotype *PARP9*** | **Genotype *NGDN*** | **Genotype *RARB*** |
| --- | --- | --- | --- | --- | --- | --- | --- | --- |
| Aisha Blue Princess | long-lived | TC | GA | GG | CC | CC | TC | TT |
| Appia Fosso Corno | long-lived | TT | GA | GA | TT | AA | TT | TC |
| Aragorn | long-lived | TC | AA | GG | CC | CC | CC | TT |
| Arena | long-lived | CC | AA | GG | TC | CC | TT | CC |
| Avalanche Crnobog | long-lived | CC | AA | GG | TC | CC | CC | X |
| Azzura Giantpaw's | long-lived | TT | GA | GA | TC | AA | TT | TT |
| Cesare | long-lived | CC | GA | GG | TC | AA | TC | TT |
| Cora | long-lived | TC | GG | GA | TT | CC | CC | CC |
| Dominika Atison | long-lived | CC | GG | GG | CC | CC | X | TT |
| Edgar Georg Cane Corso | long-lived | TC | GG | GA | TC | AA | TC | TC |
| Fee dei Fontanili | long-lived | TC | GA | GA | TT | CC | TT | CC |
| Gera | long-lived | CC | AA | GG | X | CC | TT | X |
| Gloria Abdanka Cember | long-lived | TC | X | GG | X | CC | TC | X |
| Greta Corso of Bajer | long-lived | TC | AA | GA | TT | CC | TT | TC |
| Hihankara's Eleganza | long-lived | X | GG | X | X | X | X | X |
| Irvin Corso pod Zoborom | long-lived | TC | GG | GG | X | X | X | X |
| Nico | long-lived | TC | GG | GA | CC | CC | X | TT |
| Samba | long-lived | TT | GG | GA | TC | CC | TC | TC |
| Tiana Bailando Con Lobos | long-lived | TT | GA | AA | CC | AA | TT | CC |
| Your Majesty del Orte | long-lived | TC | AA | GA | TT | CC | TT | TT |
| Aristocrat Korec Corso | reference | CC | AA | GG | TC | CC | TT | TT |
| Blú Petto Largo | reference | CC | AA | GG | TC | CC | TT | TT |
| Borgie Korec Corso | reference | CC | AA | GG | CC | CC | TT | TT |
| Boy Korec Corso | reference | CC | GA | GG | CC | CC | TC | TT |
| Brittany Korec Corso | reference | CC | X | GG | CC | CC | TT | TT |
| Bucci of Michalec | reference | TC | GG | GG | TC | X | CC | TT |
| Ceres Conny Gemma Nera | reference | CC | AA | GG | CC | CC | TT | TC |
| Chalu Gucci Angelo Custode | reference | CC | AA | GG | CC | CC | TT | TT |
| Dakar Gemma Nera | reference | TC | AA | GA | TC | CC | TC | CC |
| Elisa Berren Corso | reference | CC | AA | GG | CC | CC | CC | TT |
| Faith Petto Largo | reference | TC | GA | GG | CC | CC | TT | TT |
| Infinity Geraldino | reference | CC | AA | GG | CC | CC | TC | TT |
| Ivan Petto Largo | reference | CC | AA | GG | CC | CC | TT | TT |
| Kate Atison | reference | CC | GA | GG | CC | AA | TT | TT |
| Koleta Atison | reference | CC | GA | GG | TC | CC | TT | TT |
| Madeira Petto Largo | reference | CC | AA | GG | CC | CC | TT | TT |
| Samm Corso z Jasnej hviezdy | reference | CC | GA | GG | CC | CC | X | TT |
| Sofie Atison | reference | TC | GA | GA | TC | CC | TT | CC |
| Xavera Atison | reference | TC | GA | GA | CC | CC | TT | TC |
| Zeta Crazy Clan | reference | TC | AA | GG | CC | CC | TT | TT |

**Table A3.** Primer sequences for PCR amplification of selected regions.

| **Gene** |  | **Primer sequence** |
| --- | --- | --- |
| ***TDRP*** | Forward primer | TGACCTTTGGAAGGTTGGGTT |
|  | Reverse primer | TCTGAGGTTGCTGCATCGTG |
| ***FBOX25*** | Forward primer | CCCGTTCTTTTCACAGGGTG |
|  | Reverse primer | CAGAGAGCACATGGGGCTAA |
| ***MC2R*** | Forward primer | ACTAGGGACACTCAAGACATTTT |
|  | Reverse primer | GCCTTTGTCATCTTTAGTTCTCACA |
| ***PARP9*** | Forward primer | AAGACCTCAGGTTTCTAGCTGT |
|  | Reverse primer | CTCACAAAAACGACTTAAGGTCAGA |
| ***FBXL21*** | Forward primer | AGATGTCTGAGGGAAATGTGGAA |
|  | Reverse primer | GCAAAGTTCACTGACTGCTTCA |
| ***NGDN*** | Forward primer | CCCCTCGAGCTGACGAATTT |
|  | Reverse primer | CGACATTCCCACTCCACTCC |
| ***RARB*** | Forward primer | CCTCAGAAGAACCACCATCAA |
|  | Reverse primer | TCTGGCTCCATCCACTGACA |

**Table A4.** PCR protocol for amplification.

|  | **Temperature** | **Time** |
| --- | --- | --- |
| **Initiation** | 95°C | 2 min |
| **Denaturation** | 95°C | 20 sec |
| **Annealing** | 59°C | 30 sec |
| **Extension** | 65°C | 2 min |
| **Final extension** | 65°C | 5 min |
| **Number of cycles** |  | 33 cycles |

*
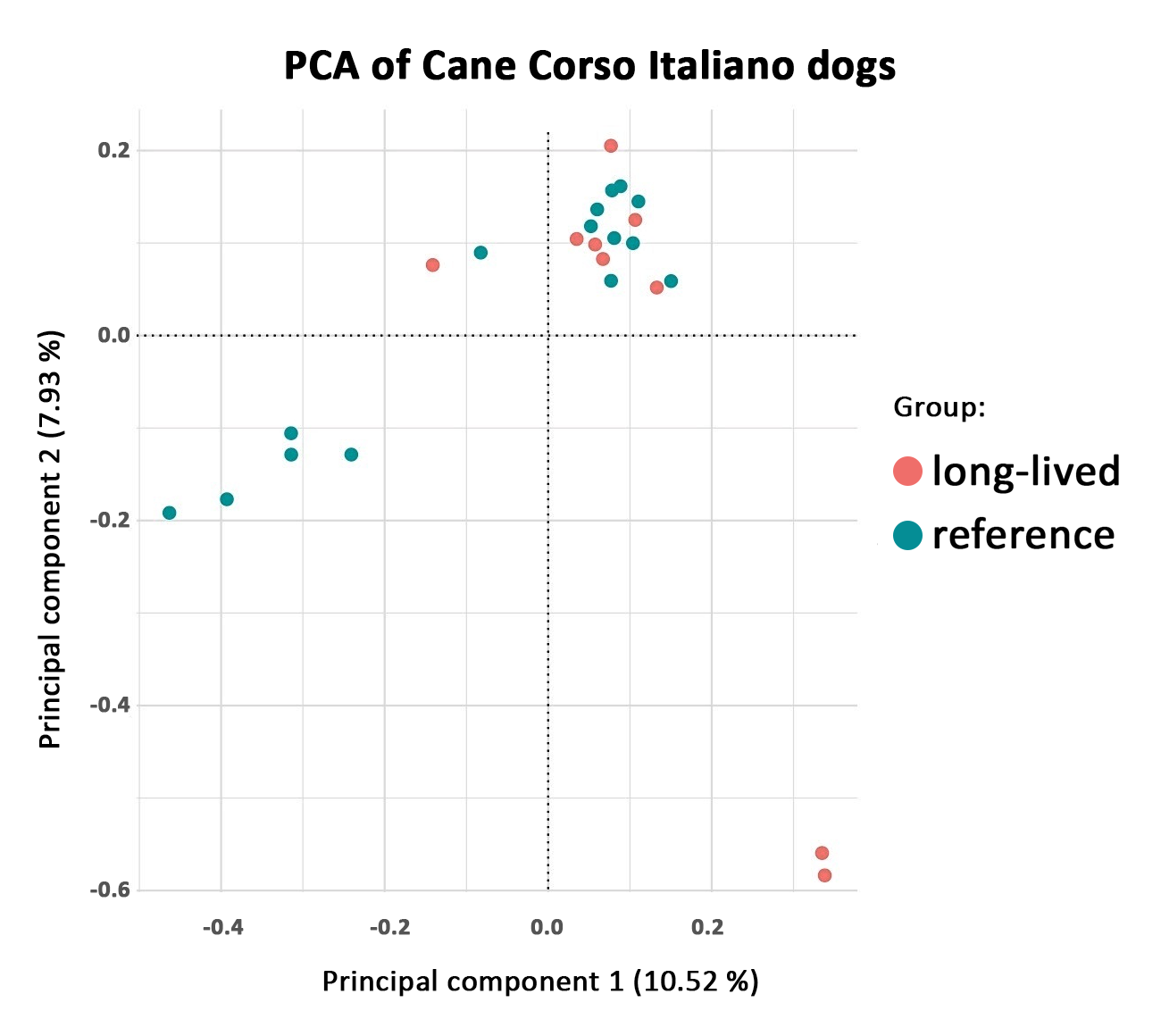
*

**Figure A1**. Principal component analysis of genotyped dog samples

Principal component analysis showed stratification of the samples (Fig. A1) that is affected by presence of closely related individuals in a small dataset. PCA should be interpreted with caution since there is a risk of false results [1]. Since GWAS was used as a prediction tool for further evaluation by sequencing and more samples were added supporting the GWAS results, all the samples were kept for the analysis.

**
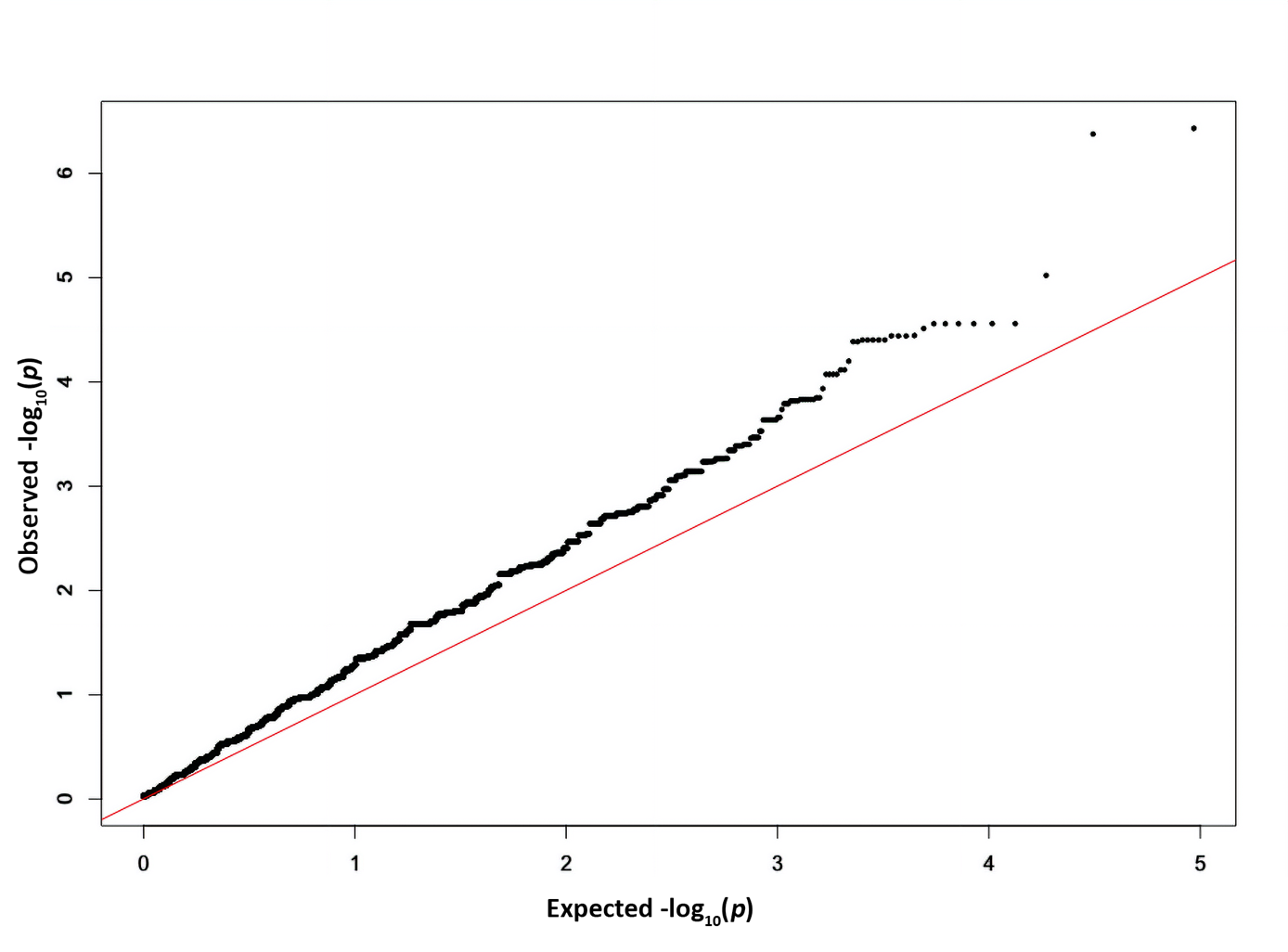
Figure A2**. Qqplot of the GWAS results. Red line represents the expected and the black dotted line represents the observed P-values


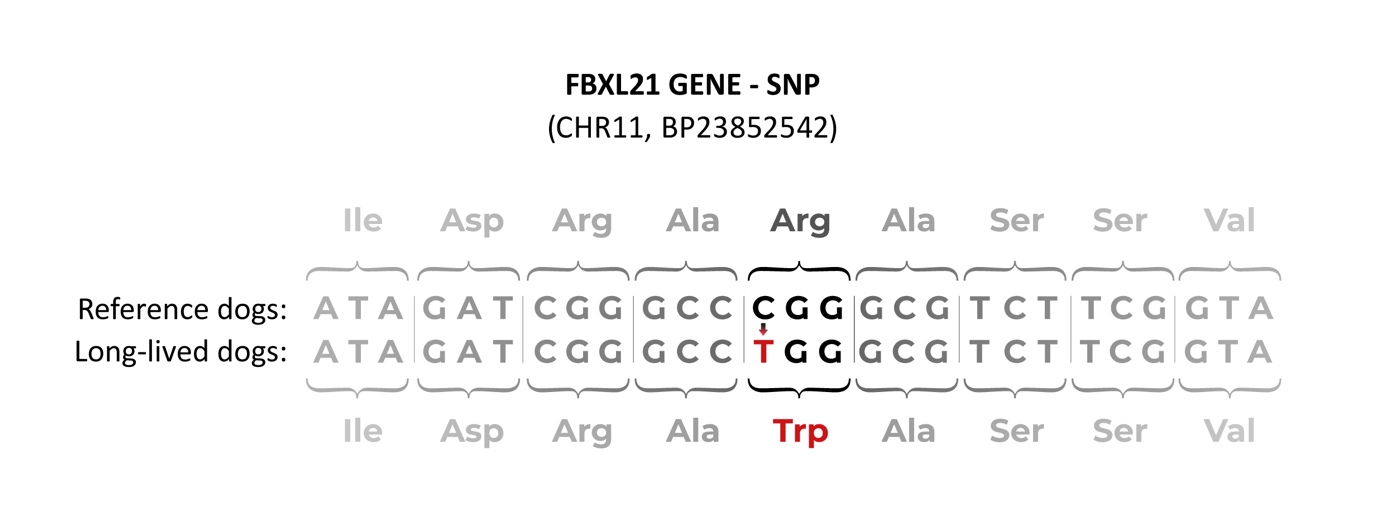


**Figure A3.** Nucleotide substitution and aminoacid change in SNP chr11_23852542 located in *FBXL21* gene.

References:

1. Björklund, M. (2019), Be careful with your principal components. Evolution, 73: 2151-2158. https://doi.org/10.1111/evo.13835
